# Exploring the link between osteoporosis and stroke risk: An exploratory study based on 2017–2018 NHANES clinical data and bioinformatics analysis

**DOI:** 10.1371/journal.pone.0337460

**Published:** 2025-12-11

**Authors:** Weimin Ren, Wen Shu, Shuzhong Huang, Yufei Qin, Juan Hu, Zhanying Shi

**Affiliations:** 1 Department of Orthopedics, Liuzhou People’s Hospital (Liuzhou People’s Hospital Affiliated to Guangxi Medical University), Liuzhou, Guangxi, China; 2 Clinical laboratory, Lianyungang Second People’s Hospital Affiliated to Kangda College of Nanjing Medical University, Lianyungang, Jiangsu, China; 3 Lianyungang Clinical College, Bengbu Medical University & The Second People’s Hospital of Lianyungang, Lianyungang, Jiangsu, China; Guangdong Nephrotic Drug Engineering Technology Research Center, Institute of Consun Co. for Chinese Medicine in Kidney Diseases, CHINA

## Abstract

**Objective:**

This study aims to explore the correlation between Osteoporosis and stroke risk, and find potential common key genes and drugs for intervention through bioinformatics methods.

**Methods:**

This study used clinical data to assess the relationship between Osteoporosis and stroke risk through univariate and multivariate logistic regression analyses. Additionally, blood sequencing data from patients with Osteoporosis and stroke were obtained from the GEO database, and common key genes were identified using differential analysis, LASSO regression, and ROC curve methods. Potential interventional drugs were predicted using the DSigDB database.

**Results:**

In the initial model, Osteoporosis was significantly associated with stroke risk (OR=1.78, 95% CI: 1.14–2.78, *p* < 0.01). This association was still significant after adjusting for factors such as age, gender, race, and BMI (OR=1.84, 95% CI: 1.18–2.89, *p* = 0.007). Bioinformatics analysis identified *LILRA5*, *HNRNPL* and *AGBL3* as common key genes for Osteoporosis and stroke, and these genes were highly effective in diagnosing both diseases. The DSigDB database predicted that Cyclopenthiazide, Neostigmine bromide, and R-atenolol could potentially intervene with these three genes.

**Conclusion:**

There is a significant positive correlation between Osteoporosis and stroke risk. *LILRA5*, *HNRNPL* and *AGBL3* could be key genes common to both diseases, and Cyclopenthiazide, Neostigmine bromide, and R-atenolol could be potential drugs for intervention.

## 1. Introduction

Osteoporosis and stroke are two common age-related diseases that create a significant burden on global health. Osteoporosis is marked by lower bone density and damage to bone structure, affecting approximately 200 million people worldwide [1]. Large cohort studies have confirmed that the risk of stroke in patients with Osteoporosis significantly increases by 24% within five years (hazard ratio = 1.24) [2]. Stroke is the second leading cause of death globally, with about 13.7 million new cases each year [3]. It’s important to note that compared to people without stroke, hemorrhagic stroke patients face a 2.06 times greater risk of Osteoporosis (95% CI: 1.83–2.33), and ischemic stroke patients face a 1.77 times greater risk (95% CI: 1.65–1.89) [4]. As the global population ages, the comorbidity burden of these two diseases will pose greater challenges to public health systems.

Low bone density has been associated with an increased risk of stroke as an independent correlated factor [5]. People with Osteoporosis often have issues with blood vessel function, and carotid ultrasound shows that high echogenic plaques (reflecting calcification) are significantly associated with Osteoporosis (odds ratio OR=6.58) [6], suggesting a potential associative link between bone metabolism disorders and vascular lesions. Osteoporosis after stroke is characterized by specific bone loss on the hemiplegic side, primarily due to reduced mechanical loading from limited limb activity. Comorbidity significantly increases disability and mortality rates. The risk of hip fracture in stroke patients is four times higher than that of the general population, with a mortality rate of 15–25% within one year post-fracture [7], creating a vicious cycle of “stroke-bone loss-fracture-recurrent stroke.”

Previous studies have mainly focused on the pathogenesis and treatment methods of osteoporosis and stroke individually [8,9], with limited research investigating their interconnection. Some studies have reported a higher prevalence of stroke in patients with osteoporosis; however, most of these studies have small sample sizes and have not adequately accounted for confounding factors. Therefore, the reliability of their conclusions requires further verification. Additionally, many studies have only analyzed stroke as a risk factor for osteoporosis, neglecting the bidirectional relationship between the two conditions [10]. Furthermore, few studies have explored the potential connections between osteoporosis and stroke from a molecular biology perspective, lacking the identification of common key genes and the investigation of pharmacological interventions.

**Fig 1:**
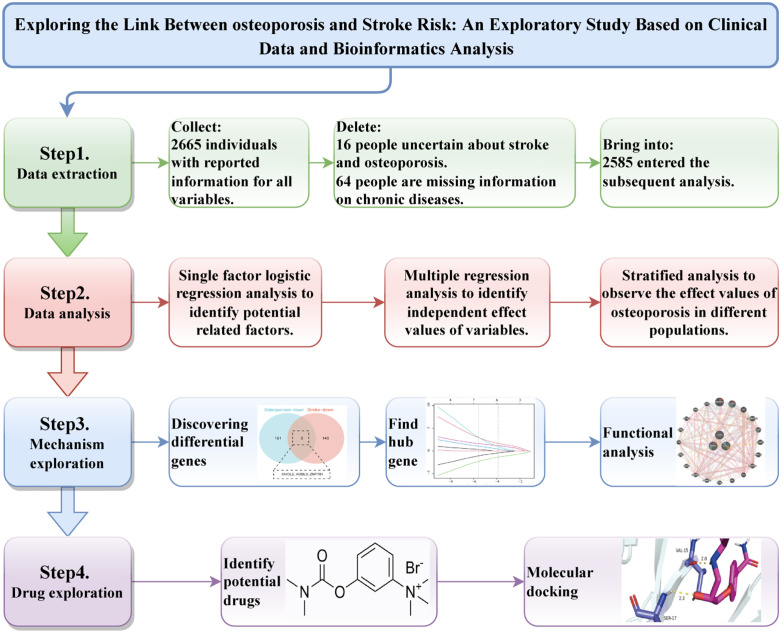
Research flow chart.

Therefore, this study integrates large-scale clinical data with multi-layered bioinformatics analyses to clarify the association between Osteoporosis and stroke risk, identify shared key genes and their mechanisms, and predict potential therapeutic agents. These findings are expected to provide new insights for the prevention and treatment of Osteoporosis–stroke comorbidity and to establish a foundation for future translational research. The research process is shown in Fig 1.

## 2. Methods

### 2.1. Data sources and inclusion/exclusion criteria

The research data comes from the 2017–2018 cycle of the National Health and Nutrition Examination Survey (NHANES) database (https://www.cdc.gov/nchs/nhanes/). NHANES employs a stratified multistage probability sampling design, ensuring national representativeness. Inclusion criteria: ① Age ≥ 20 years; ② Complete data on key variables, specifically Osteoporosis and stroke. Exclusion criteria: ① Age < 20 years; ② Incomplete reporting of inclusion criteria. NHANES has been approved by the NCHS Research Ethics Review Board (https://www.cdc.gov/nchs/nhanes/irba98.htm).

The Gene Expression data used in this study were all derived from the Gene Expression Omnibus (GEO) database under the National Center for Biotechnology Information (NCBI) of the United States. According to the consistency of the research topic and sample source (blood), relevant datasets related to osteoporosis and stroke were screened. The specific information is as follows

The GSE56116 dataset was selected as the data source for the analysis of gene expression related to osteoporosis, including the gene expression profile data of 10 patients with osteoporosis and 3 healthy controls. Racial distribution: The samples are mainly derived from Asian populations; Sequencing platform: GPL4133 (Agilent-014850 Whole Human Genome Microarray 4x44K G4112F, Feature Number version); Original data format: CEL file.

The GSE58294 dataset was selected as the data source for the analysis of gene expression related to stroke. Racial distribution: The original submitted documents and annotation information of the dataset did not clearly record the racial background of the samples. Sequencing platform: GPL570 (Affymetrix Human Genome U133 Plus 2.0 Array); Original data format: CEL file.

Data preprocessing scheme: Appropriate standardized methods are adopted for different sequencing platforms, among which GSE56116 (Agilent platform) uses the quantile standardized method. GSE58294 (Affymetrix platform) uses the normalizeBetweenArrays function of the limma package for standardization to eliminate batch effects and detection system errors among samples, making the gene expression distribution of each sample tend to be consistent.

Secondly, to reduce background noise interference, a uniform low expression threshold (log2(expression) < 5) was set. Probes with expression values lower than this threshold in all included samples were filtered out, and only high-expression probes with potential biological significance were retained for subsequent analysis.

Finally, the probe ID is converted into the corresponding gene symbol through the probe annotation file. In the case where multiple probes correspond to the same gene, the average expression value is taken as the final expression value of the gene.

### 2.2. Data collection of study subjects

Data collection for study subjects includes several aspects: first, demographic factors like age, gender, race, and Body Mass Index (BMI); second, the history of metabolic diseases including hypertension and diabetes; as well as cardiovascular history including heart failure and angina; finally, laboratory indicators such as cholesterol, serum glucose, and triglycerides.

Definition of Osteoporosis: For survey participants, based on the indicator OSQ060, the question is: “Has a doctor ever told {you/SP} that {you/s/he} had Osteoporosis, sometimes called thin or brittle bones?” Participants answering “yes” are defined as having Osteoporosis.

Definition of stroke: For survey participants, based on the indicator MCQ160F, the question is: “Has a doctor or other health professional ever told {you/SP} that {you/s/he}... had a stroke?” Participants answering “yes” are defined as having had a stroke. The NHANES database does not include classification information for stroke subtypes (ischemic/hemorrhagic), and only records the overall situation of ‘stroke history’. Therefore, the multiple regression analysis and stratified analysis in this study cannot be further refined according to stroke subtypes.

All information is collected. Blood samples are taken at the Mobile Examination Center (MEC), basic information is immediately organized, and serum samples are sent to the National Center for Environmental Health Laboratory Science, the Centers for Disease Control and Prevention, and designated authorized agencies for scientific storage management before analysis.

### 2.3. Cross-sectional analysis

Before formal analysis, in order to address the imbalance between the Osteoporosis group and the non-Osteoporosis group in the original sample, we used propensity score matching (PSM) for sample matching [11]. The specific steps are as follows: first, Age, Gender, Race, and BMI were selected as matching variables; second, we performed 1:2 nearest-neighbor matching with a caliper value of 0.2, excluding samples outside the propensity score overlap area during the matching process; finally, after matching, intergroup balance was evaluated using standardized mean difference (SMD). If SMD < 0.1, it was considered that there was no statistically significant difference between the groups, indicating adequate comparability of the matched samples. Cross-sectional analysis primarily employs univariate and multivariate logistic regression analyses to assess the relationship between Osteoporosis and stroke risk. In univariate analysis, stroke is the dependent variable and Osteoporosis is the independent variable, calculating HR values and 95% CI. In multivariate regression analysis, we adjust for different factors step by step to construct multiple models to remove potential confounding factors, and OR values and 95% CI are calculated. Among them, Model 1 is unadjusted, Model 2 adjusts for demographic factors, Model 3 adjusts for demographic factors, metabolic diseases, and cardiovascular diseases, and Model 4 adjusts for demographic factors, metabolic diseases, cardiovascular diseases, and laboratory indicators.

### 2.4. Bioinformatics analysis and mechanism exploration

Differential expression analysis (|logFC| > 1, FDR < 0.05) of GEO datasets GSE56116 (Osteoporosis) and GSE58294 (stroke) was performed using Limma [12]. Key genes were identified by Least Absolute Shrinkage and Selection Operator (LASSO) regression (glmnet) and validated via Receiver Operating Characteristic (ROC) analysis (pROC) [13,14]; When conducting the screening of key genes, the Log Lambda value (λ.min) corresponding to minimizing the cross-validation error is selected as the optimal penalty parameter. An AUC value of 0.7 or above under the ROC curve indicates that the diagnostic ability of the model is relatively good. Finally, a Protein-Protein Interaction (PPI) network was constructed in GeneMANIA (STRING>0.4), followed by GO [15] and Kyoto Encyclopedia of Genes and Genomes (KEGG) enrichment (Cluster Profiler, *p* < 0.05) [16,17].

### 2.5. Identification of potential drugs and molecular docking

We predict potential drugs that might target key genes using the DSigDB database (http://tanlab.ucdenver.edu/DSigDB) [18], with False Discovery Rate (FDR) < 0.05 as the screening criterion. The DSigDB database is implemented using the Enrichr platform (https://maayanlab.cloud/enrichment/), which has been widely used to display multiple visual details of gene clustering functions [19]. Then, molecular docking is performed using AutoDock Vina, docking potential drugs with key genes to assess their binding stability and binding energy, with a binding energy ≤ −7.0 kcal/mol as the high affinity standard.

The protein structures used in this study include three types

The structure of AGBL3 is derived from the AlphaFold prediction model (AF-Q8NEM8-F1), which covers the full length and has a high overall confidence level, making it suitable for molecular docking research. The structure of HNRNPL is derived from the PDB database (PDB ID: 7EVR), which is the crystal structure analyzed experimentally. The X-ray diffraction method was adopted, with A resolution of 1.8 A. The structure of LILRA5 is also derived from PDB (ID: 2D3V), analyzed by X-ray diffraction method, with A resolution of 1.85 A. All structures were preprocessed by AutoDockTools before use, including water molecule removal, hydrogenation, and repair of missing residues, to ensure their integrity and suitability for subsequent docking analysis.

Ligand pretreatment: The structures of three ligand small molecules, Cyclopenthiazide, Neostigmine and R-atenolol, were all obtained from the PubChem database and were initially in SDF file format. Three-dimensional structure generation was carried out using Open Babel, and preprocessing steps such as energy minimization, hydrogenation, and charge calculation (Gasteiger charge) were completed through AutoDockTools to ensure that the ligand structure was reasonable and suitable for docking calculation.

Binding mode analysis results: AGBL3 binds to Cyclopenthiazide: The ligand binding sites involve SER-149 and AP-819, forming key interactions, and the binding conformation is stable. The binding of HNRNPL to Neostigmine: The ligand forms an important interaction with the GLY-420 residue, and the binding mode is reasonable. LILRA5 binds to R-atenolol: The ligand interacts with the residues of SER-17 and VAL-15, with clear binding sites and stable conformations.

All the above-mentioned combination modes were analyzed through the visualization tool (PyMOL), with key residues participating in hydrogen bonds or hydrophobic interactions, providing a structural basis for subsequent functional studies.

### 2.6. Statistical analysis

All data are analyzed using R version 4.1.3, with continuous variables described in detail and a 95% confidence interval. Categorical variables are represented by counts and weighted percentages. Skewed distributions are based on medians and Q1-Q3. Normal distributions are described using means and standard deviations. Based on the normality of the distribution, Student’s t-test or Mann-Whitney U test is used for inter-group comparisons of continuous variables, and Fisher’s exact probability method is used for intra-group comparisons. Univariate and multivariate logistic regression analyses are used to assess the relationship between Osteoporosis and stroke risk. Differential analysis, LASSO regression, and ROC curves are used for bioinformatics analysis. We consider a p-value < 0.05 to be statistically significant.

### 2.7. Ethics approval and consent to participate

Due to the fact that all participants involved in NHANES and GEO have agreed to investigate and signed written consent forms, they have obtained review and approval from the ethics review committee. As a publicly accessible database, no additional ethical approval is required.

## 3. Results

### 3.1. Description of the study population

A total of 2585 subjects were included in this study, divided into two groups based on the presence of Osteoporosis (Supplementary Table 1): the Non-Osteoporosis group (N = 2256) and the Osteoporosis group (N = 329). After conducting PSM analysis, a total of 951 were included (Table 1): the Non-Osteoporosis group (N = 622) and the Osteoporosis group (N = 329). There were statistically significant differences between the two groups in terms of hypertension, congestive heart failure, and stroke (*p* < 0.05). The Osteoporosis group was Higher prevalence rates of hypertension, congestive heart failure, and stroke. Among biochemical indicators, the Osteoporosis group had higher cholesterol (*p* < 0.01), However, there was no statistically significant difference between the two groups in other biochemical indicators. This indicates that patients with Osteoporosis often have a higher incidence of hypertension and cardiovascular problems, suggesting that Osteoporosis may be an important identification marker for high-risk stroke populations based on this observed association.

**Table 1:** Research population description.

Characteristic	No-Osteoporosis (N = 622)	Osteoporosis (N = 329)	P-value
Age, years	69.17 ± 8.62	69.42 ± 8.63	0.698
Gender, %			0.505
Female	519 (83.44%)	280 (85.11%)	
Male	103 (16.56%)	49 (14.89%)	
BMI, kg/m^2^	29.50 ± 6.88	29.25 ± 7.37	0.307
Race/Ethnicity, %			0.86
Mexican American	50 (8.04%)	28 (8.51%)	
Other hispanic	55 (8.84%)	32 (9.73%)	
Non-hispanic white	281 (45.18%)	155 (47.11%)	
Non-hispanic black	127 (20.42%)	58 (17.63%)	
Other	109 (17.52%)	56 (17.02%)	
Hypertension, %			0.003
No	277 (44.53%)	114 (34.65%)	
Yes	345 (55.47%)	215 (65.35%)	
Diabetes, %			0.208
No	462 (74.28%)	227 (69.00%)	
Yes	133 (21.38%)	83 (25.23%)	
Borderline	27 (4.34%)	19 (5.78%)	
Congestive heart failure, %			0.011
No	593 (95.34%)	300 (91.19%)	
Yes	29 (4.66%)	29 (8.81%)	
Angina pectoris, %			0.138
No	599 (96.30%)	310 (94.22%)	
Yes	23 (3.70%)	19 (5.78%)	
Cholesterol (mg/dL)	198.59 ± 45.57	191.00 ± 43.15	0.009
Serum glucose (mg/dL)	108.75 ± 44.35	103.96 ± 30.99	0.175
Triglyceride (mmol/L)	145.58 ± 83.13	139.06 ± 72.22	0.164
Stroke, %			0.01
No	576 (92.60%)	288 (87.54%)	
Yes	46 (7.40%)	41 (12.46%)	

### 3.2. Cross-sectional analysis of Osteoporosis and stroke

To explore the strength of the association between Osteoporosis and stroke, and to validate this relationship while adjusting for confounding factors and stratifying the population, the univariate analysis in Table 2 revealed a significant link between Osteoporosis and stroke risk (OR=1.78, 95% CI: 1.14–2.78, *p* < 0.01).

**Table 2:** Screening variables related to stroke through univariate logistic regression.

	Statistics	OR (95%CI)	P-value
Osteoporosis			
No	622 (65.40%)	Reference	
Yes	329 (34.60%)	1.78 (1.14, 2.78)	0.01
Gender, %			
Female	799 (84.02%)	Reference	
Male	152 (15.98%)	0.66 (0.33, 1.31)	0.233
Age, years	69.25 ± 8.62	1.03 (1.01, 1.06)	0.0165
Race/Ethnicity, %			
Mexican American	78 (8.20%)	Reference	
Other hispanic	87 (9.15%)	0.89 (0.25, 3.20)	0.8586
Non-hispanic white	436 (45.85%)	1.56 (0.60, 4.07)	0.3666
Non-hispanic black	185 (19.45%)	2.39 (0.88, 6.47)	0.0869
Other	165 (17.35%)	0.84 (0.27, 2.60)	0.7656
BMI, kg/m^2^	29.42 ± 7.05	1.01 (0.98, 1.04)	0.4163
Hypertension, %			
No	391 (41.11%)	Reference	
Yes	560 (58.89%)	4.43 (2.42, 8.10)	<0.001
Diabetes, %			
No	689 (72.45%)	Reference	
Yes	216 (22.71%)	1.79 (1.12, 2.87)	0.015
Borderline	46 (4.84%)	0.00 (0.00, Inf)	0.979
Congestive heart failure, %			
No	893 (93.90%)	Reference	
Yes	58 (6.10%)	5.37 (2.93, 9.87)	<0.001
Angina pectoris, %			
No	909 (95.58%)	Reference	
Yes	42 (4.42%)	2.91 (1.34, 6.29)	<0.001
Cholesterol (mg/dL)	195.96 ± 44.87	0.99 (0.99, 1.00)	0.024
Serum glucose (mg/dL)	107.10 ± 40.28	1.00 (1.00, 1.01)	0.668
Triglyceride (mmol/L)	143.32 ± 79.55	1.00 (1.00, 1.00)	0.868

To further clarify the independent relationship between the two, multivariate regression analysis was conducted (Table 3). In Model 1 (unadjusted), Osteoporosis was significantly associated with stroke risk (OR=1.78, 95% CI: 1.14–2.78, *p* = 0.01). In Model 2, after adjusting for factors such as age, gender, race, and BMI, this association remained (OR=1.76, 95% CI: 1.18–2.63, *p* = 0.005). In Model 3, after further adjusting for chronic diseases (OR=1.54, 95% CI: 1.02, 2.33, *p* = 0.040) and in Model 4, after adjusting for biochemical indicators and other confounding factors (OR=1.52, 95% CI: 1.00, 2.30, *p* = 0.047), the effect was less pronounced but the trend persisted.

**Table 3:** Multiple regression analysis of Osteoporosis and stroke.

Exposure	Model 1, OR (95%CI) P-value	Model 2, OR (95%CI) P-value	Model 3, OR (95%CI) P-value	Model 4, OR (95%CI) P-value
Osteoporosis				
No	Reference	Reference	Reference	Reference
Yes	1.78 (1.14, 2.78) 0.010	1.84 (1.18, 2.89) 0.007	1.69 (1.06, 2.70) 0.026	1.66 (1.04, 2.65) 0.034

Model 1 adjust for: None.

Model 2 adjust for: Age;Gender;Race;BMI.

Model 3 adjust for: Age;Gender;Race;BMI;Hypertension;Diabetes;Congestive heart failure;Angina pectoris.

Model 4 adjust for: Age;Gender;Race;BMI;Hypertension;Diabetes;Congestive heart failure;Angina pectoris;Cholesterol;Serum glucose;Triglyceride

**Fig 2:**
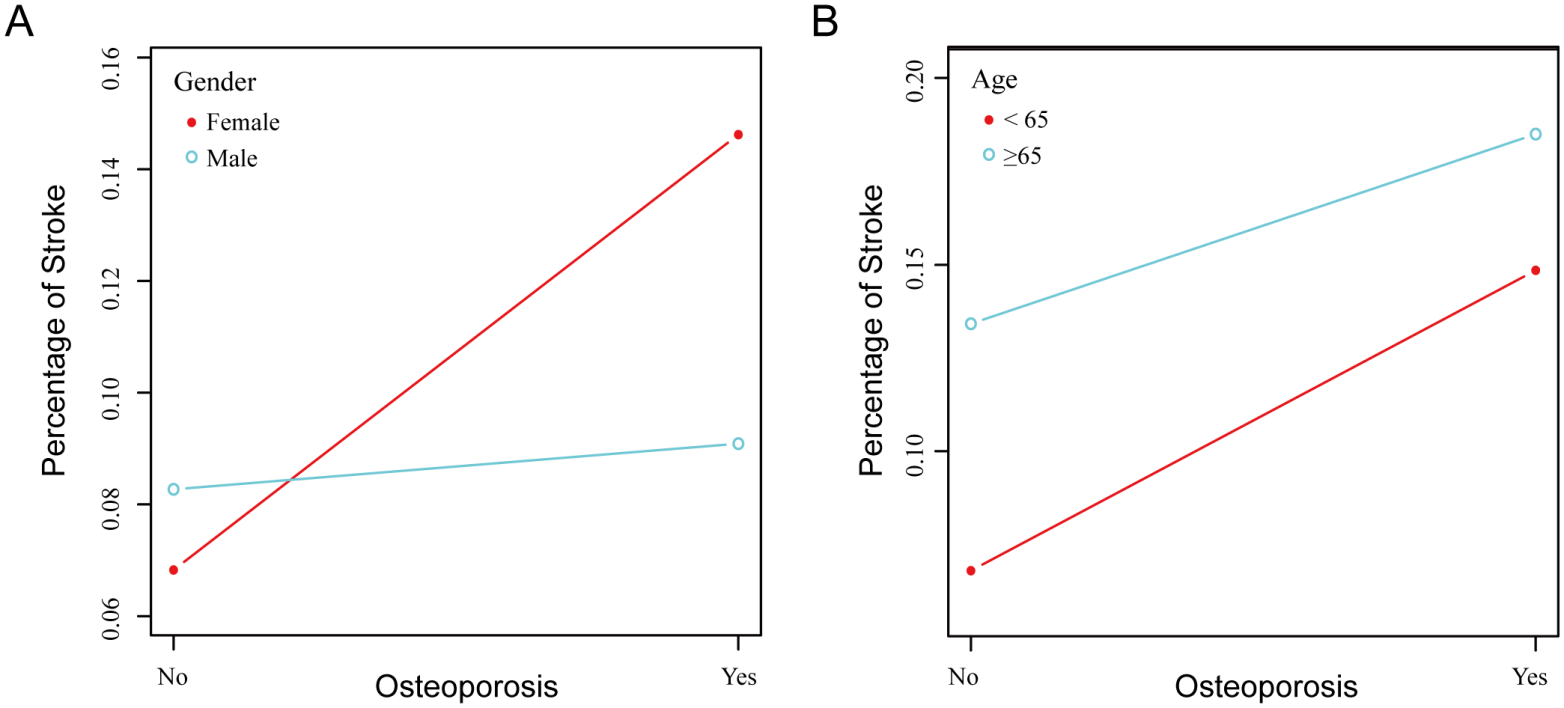
The relationship between Osteoporosis and stroke was analyzed by stratification. (A) Stratified observation of correlation by gender. (B) stratified observation of correlation by age.

Finally, stratified analysis showed that Osteoporosis was positively associated with stroke in both genders and across different age groups (Fig 2A and B), indicating that this association does not have significant gender or age interaction effects.

In each graph, the X axis represents the presence or absence of Osteoporosis, and the Y axis represents the percentage of stroke. The red and blue lines/dots represent their respective subgroups.

### 3.3. Identification of key genes and functional exploration

**Fig 3:**
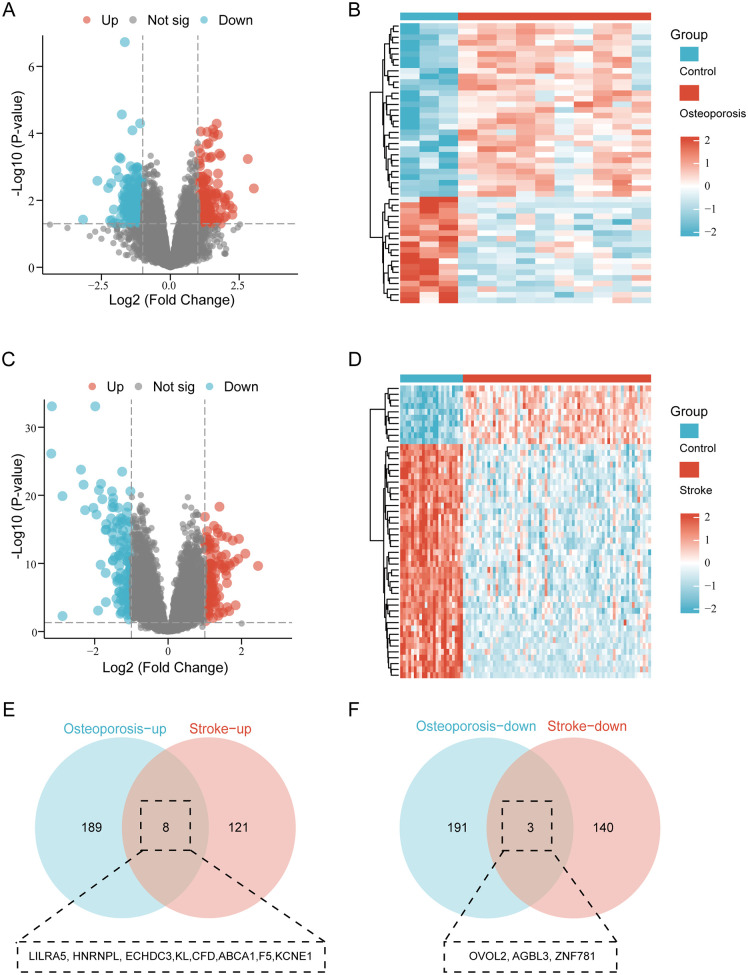
Differential gene expression and overlap analysis. (A, B) Volcano plots and heat map showing differentially expressed genes (DEGs) in peripheral blood of Osteoporosis. (C, D) Volcano plots and heat map showing differentially expressed genes (DEGs) in peripheral blood of stroke. (E) Venn diagram illustrating the overlap of up-DEGs between Osteoporosis and stroke. (F) Venn diagram illustrating the overlap of down-DEGs between Osteoporosis and stroke.

To shed light on the shared molecular mechanisms of both diseases, differential analysis was performed on peripheral blood sequencing data from Osteoporosis and stroke patients in the GEO database. A total of 391 differentially expressed genes were identified in the Osteoporosis group (Fig 3A and B). Meanwhile, 272 differentially expressed genes were identified in the stroke group through the same differential analysis criteria (Fig 3C and D). Analyzing the overlap of differentially expressed genes with a Venn diagram revealed 11 common differentially expressed genes, with 8 upregulated and 3 downregulated (Fig 3E and F).

**Fig 4:**
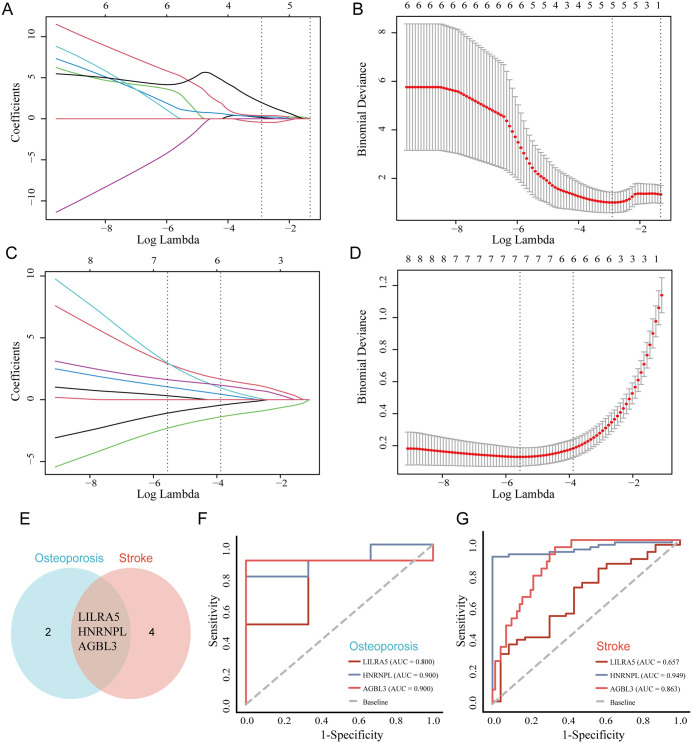
LASSO regression screening and ROC evaluation of shared key genes. (A–D) LASSO coefficient profiles and cross-validation plots for selecting key genes in Osteoporosis (A, B) and stroke (C, D). Dotted vertical lines indicate the optimal λ values (log λ) that yield the minimum mean-squared error. (E) Venn diagram showing the intersection of key genes identified from LASSO models of Osteoporosis and stroke, resulting in *LILRA5*, *HNRNPL* and *AGBL3* as common hub genes. (F, G) ROC curves demonstrating the diagnostic performance of *LILRA5*, *HNRNPL* and *AGBL3* in Osteoporosis (F) and stroke (G).

In order to identify as many comorbidity related genes as possible, LASSO regression analysis was performed on the 11 common genes in two datasets for dimensionality reduction. Select the minimum Log Lambda value corresponding to the maximum number of genes as the optimal choice, 5 key genes were selected from the Osteoporosis group (Fig 4A and B), while 6 key genes were selected from the stroke group (Fig 4C and D). After intersecting the results from both groups, *LILRA5, HNRNPL* and *AGBL3* were identified as common key genes (Fig 4E). Meanwhile, diagnostic ROC curves showed that, except for *LILRA5* which had moderate diagnostic efficacy, *HNRNPL* and *AGBL3* both exhibited high diagnostic efficacy in Osteoporosis and stroke, with area under the curve (AUC) values greater than 0.85 (Fig 4F and G), More relevant indicators for diagnosing AUC, such as Sensitivity, Specificity, Youden index, Optimal threshold, and Accuracy, are shown in Table 4.

**Table 4:** Diagnostic efficacy indicators of four key genes in diseases.

Feature	AUC value	Sensitivity	Specificity	Youden index	Optimal threshold	Accuracy
Osteoporosis						
LILRA5	0.800	0.900	0.667	0.567	5.838	0.231
HNRNPL	0.900	0.800	1	0.800	7.440	0.231
AGBL3	0.900	0.900	1	0.900	1.801	0.231
Stroke						
LILRA5	0.657	0.710	0.565	0.275	−2.568	0.663
HNRNPL	0.949	0.899	1	0.899	0.813	0.913
AGBL3	0.863	0.957	0.667	0.623	−4.210	0.272

**Fig 5:**
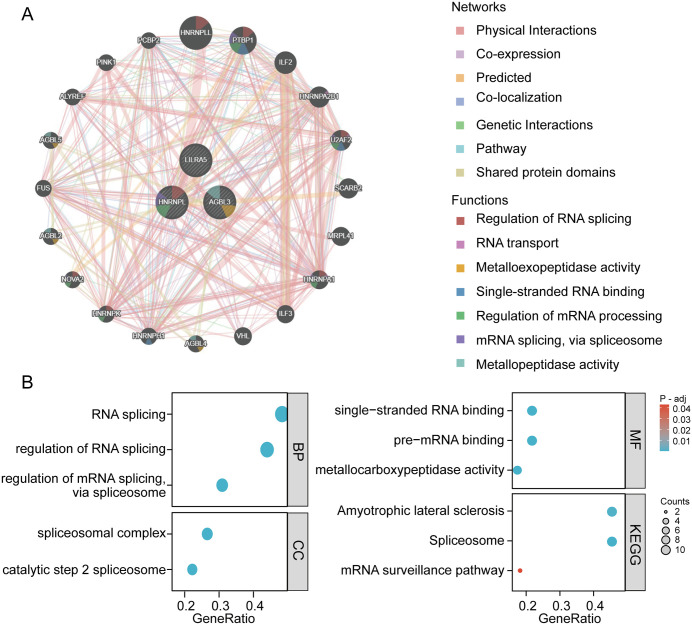
Functional enrichment analysis of the shared key-gene network. (A) Protein–protein interaction (PPI) network of the three hub genes (*LILRA5*, *HNRNPL*, *AGBL3*) and their 20 first-order interacting partners constructed in Gene MANIA. Edge types denote physical interactions, co-expression, co-localization, predicted relationships, shared protein domains, and genetic interactions. (B) Gene Ontology (GO) and KEGG pathway enrichment results. Bubble plots illustrate significant biological processes (e.g., regulation of RNA splicing, metallopeptidase activity) and pathways (Spliceosome, mRNA surveillance pathway, Amyotrophic lateral sclerosis).

To elucidate the potential mechanisms of *LILRA5*, *HNRNPL* and *AGBL3* in Osteoporosis and stroke, a protein-protein interaction (PPI) network was constructed using GeneMANIA, including 3 core genes and their 20 direct interacting partners, forming a functional network with 23 nodes (Fig 5A). The network edge types were primarily physical interactions (77.6%) and co-expression (8.0%). Functional enrichment analysis of the 23 genes showed that the network was significantly enriched in RNA splicing regulation (FDR = 0.01), single-stranded RNA binding (FDR = 0.02), and metallopeptidase activity (FDR = 0.03); KEGG pathway analysis further pointed to key pathways such as “spliceosome” (hsa03040, Gene Ratio = 0.41), “mRNA surveillance pathway” (hsa03015, Gene Ratio = 0.35), and “amyotrophic lateral sclerosis” (hsa05014, Gene Ratio = 0.26) (Fig 5B). These results suggest that the aforementioned three genes may play a synergistic role in the comorbidity of Osteoporosis and stroke by regulating RNA splicing and metallopeptidase activity, thereby affecting the homeostasis of the neuro-bone metabolic axis.

### 3.4. Identification of potential drugs and molecular docking

Potential drugs that may intervene with the key genes were predicted using the DSigDB database (Table 5), specifically Cyclopenthiazide (intervening *AGBL3*), Neostigmine bromide (intervening *HNRNPL*), and R-atenolol (intervening *LILRA5*).

**Table 5:** Candidate drug predicted using DSigDB.

Term	Clinical applications or potential	Mechanism of action	P-value	Genes
Cyclopenthiazide	Promote the excretion of sodium and water, reduce blood volume and blood pressure.	Reduce the reabsorption of sodium and chlorine and increase their excretion.	0.040	AGBL3
Neostigmine bromide	Enhance neuromuscular conduction, improve muscle weakness, and promote smooth muscle contraction.	Enhance stimulation of nicotinic and muscarinic receptors, promote neuromuscular conduction and muscle contraction.	0.010	HNRNPL
R-atenolol	Reduce heart rate and myocardial contractility, and decrease cardiac oxygen consumption.	Inhibit sympathetic nervous system excitation, reduce heart rate, myocardial contractility, and conduction velocity.	0.020	LILRA5

**Fig 6:**
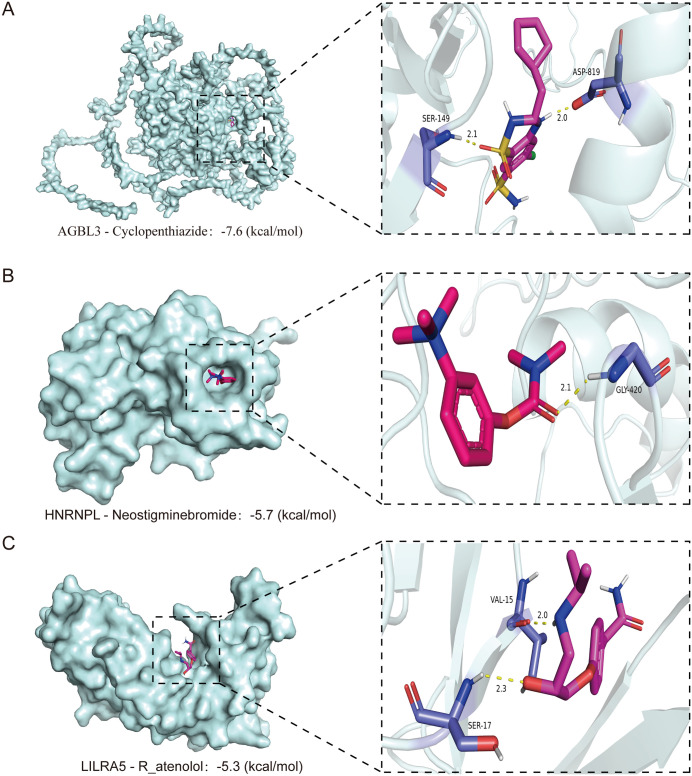
Molecular docking of predicted repositioned drugs with shared key proteins. (A) Cyclopenthiazide docked to *AGBL3*, forming hydrogen bonds (yellow dashes) with Ser-149 and yielding a binding energy of –7.6 kcal/mol. (B) Neostigmine bromide bound to *HNRNPL*, interacting with Ser-17 and exhibiting a binding energy of –5.7 kcal/mol. (C) R-atenolol positioned in *LILRA5*, with key contacts leading to a binding energy of –5.3 kcal/mol.

To validate the molecular binding capabilities of the three candidate drugs selected from DSigDB with the proteins encoded by the key genes related to Osteoporosis-stroke comorbidity (*AGBL3*, *HNRNPL*, *LILRA5*), to provide a structural basis for subsequent in vitro/in vivo functional validation and drug repositioning, molecular docking was performed on *AGBL3*, *HNRNPL*, and *LILRA5* using AutoDock Vina. The results showed that Cyclopenthiazide had a binding energy of −7.6 kcal/mol with AGBL3 (Fig 6A), Neostigmine bromide had a binding energy of −5.7 kcal/mol with HNRNPL (Fig 6B), and R-atenolol had a binding energy of −5.3 kcal/mol with LILRA5 (Fig 6C), all below the stability threshold of −5 kcal/mol. All three drugs embedded into the active pockets of the target proteins through hydrogen bonds and hydrophobic interactions, indicating their potential for in vitro binding affinity and serving as candidate molecules for subsequent functional validation and drug repositioning.

## 4. Discussion

This study is significantly different from other similar studies because it adjusts for multiple potential confounding factors. It confirms that an independent positive correlation exists between osteoporosis and the stroke risk (OR=1.66, 95% CI: 1.04–2.65, p = 0.034). However, the specific molecular pathways that connect Osteoporosis (a bone metabolic disorder) to stroke (a vascular disease) remain unclear—this gap limits our understanding of the mechanisms underlying their association and the development of targeted interventions.

After establishing the independent association between Osteoporosis and stroke, we next explored their shared molecular mechanisms. Specifically, this observed association is closely tied to the co-occurrence of vascular calcification and endothelial dysfunction in patients with Osteoporosis [6], though the directional relationship between these factors remains unclear. Although metabolic diseases (hypertension/diabetes) and biochemical indicators (such as blood glucose, lipids) contribute to comorbidity risk, Osteoporosis still exhibits pathogenic effects independent of classical metabolic disorders [20–22]. This finding adds new evidence to the concept of the “bone-vascular comorbidity axis”: in addition to the known triad of aging-inflammation-metabolic dysregulation, Osteoporosis is potentially associated with elevated stroke risk, and this association may involve the presence of vascular calcification and neurovascular unit damage, though direct regulatory effects cannot be confirmed. It suggests that Osteoporosis is not only a secondary manifestation of aging or metabolic diseases but also an independent correlate predictive of vascular lesions (based on observed associations), warranting exploration of its potential role in stroke risk stratification rather than direct causal targeting for prevention.

Based on the aforementioned association, we identified *LILRA5*, *HNRNPL*, and *AGBL3* as common hub genes for Osteoporosis and stroke, which have been externally validated in GEO transcriptomic data (AUC > 0.8) and have the potential to be developed as low-cost qPCR or liquid biopsy biomarkers. After identifying *LILRA*5, *HNRNPL*, and *AGBL3* as common hub genes for Osteoporosis and stroke, their synergistic regulatory mechanisms further confirm the molecular basis of comorbidity. *HNRNPL*, as a core regulator of RNA splicing, links osteogenic gene dysfunction and neurovascular unit damage: its abnormal splicing regulation of *RUNX2* and *COL1A1* directly leads to reduced bone formation, while its impact on *BDNF* and VE-cadherin splicing destroys cerebrovascular stability, forming a “splicing disorder-mediated dual damage” pattern. This is consistent with the findings of [23,24], which verified the key role of HNRNPL in bone-vascular crosstalk through cell models and animal experiments. *AGBL3* mediates the “inflammation-osteoclastic resorption-vascular calcification” axis through metallopeptidase activity, which is a key bridge connecting bone loss and vascular lesions. The degradation of OPG by *AGBL3* accelerates osteoclastic resorption, while the hydrolysis of OPN promotes vascular calcification—two processes that are highly consistent with the comorbid characteristics of Osteoporosis and stroke (e.g., vascular calcification in Osteoporosis patients and bone loss in stroke patients) [6,25]. The high binding affinity between Cyclopenthiazide and *AGBL*3 suggests that targeting *AGBL3* may simultaneously improve bone metabolism and vascular calcification, providing a potential direction for comorbidity intervention. *LILRA5* amplifies the dual damage effect through inflammatory regulation: the TNF-α/IL-1β released by its activated monocytes not only directly promotes inflammation in bone and vascular tissues, but also regulates the functions of *HNRNPL* and *AGBL3*, forming a “inflammation-driven functional cascade” [26]. This cascade effect explains why inflammatory factors (e.g., *TNF-α*, *IL-1β*) are commonly elevated in both Osteoporosis and stroke patients, and also provides a molecular basis for the “inflammation as a common pathological basis” hypothesis of comorbidity. In summary, the three key genes synergistically affect bone metabolism and cerebrovascular homeostasis through “RNA splicing regulation” and “metallopeptidase activity”, directly linking gene functions to comorbid phenotypes. This mechanism not only supports the reliability of the key genes identified in this study, but also provides a new theoretical basis for understanding the “bone-vascular comorbidity axis”.

Our bioinformatics analysis revealed that the interaction network composed of *LILRA5*, *HNRNPL* and *AGBL3* was significantly enriched in functions such as’ RNA splicing regulation ‘and’ metal peptidase activity ‘, and was closely related to the ‘amyotrophic lateral sclerosis pathway (hsa05014)’. This indicates that the three may mediate the comorbidity mechanism of Osteoporosis and stroke through a synergistic molecular network rather than acting independently. The ALS pathway, as a typical neurodegenerative disease pathway, involves neuronal apoptosis, neuroinflammation and oxidative stress at its core [27]. The regulatory effects of the core genes of the ALS pathway (*SOD1*, *FUS*, *TARDBP*) on ROS metabolism and inflammatory responses are common pathological drivers of Osteoporosis and stroke. For instance, the accumulation of ROS caused by abnormal *SOD1* function not only aggravates bone loss by inhibiting osteoblast differentiation and promoting osteoclast activation, but also participates in the progression of stroke by destroying cerebral vascular endothelial cells and expanding infarction area [28]. This indicates that the enrichment of the ALS pathway in this study is not a random result, but reflects the core pathological mechanism shared by Osteoporosis, stroke and ALS – namely the “oxidative stress-neuroinflammation” axis.

Based on these molecular hubs, this study proposes three translational pathways: ① Develop early diagnostic markers for Osteoporosis-stroke comorbidity based on gene expression profiles such as *LILRA5*; ② Shorten the R&D cycle through repositioning old drugs (e.g., Cyclopenthiazide targeting vascular calcification and bone metabolism); ③ Design combination therapies targeting *AGBL3*/*HNRNPL* to achieve “one drug for two diseases.” Subsequent research could expand target exploration through multi-omics integration (epigenome + proteome) and validate cross-organ efficacy using organoid models.

The innovations of this research are mainly reflected in the following three aspects: ① For the first time, a large sample of NHANES clinical data (2,585 subjects, 951 after PSM) was combined with multi-level bioinformatics analysis (differential expression analysis, LASSO regression, ROC analysis, PPI network, GO/KEGG enrichment analysis, molecular docking). Systematically exploring the relationship between osteoporosis and stroke from both clinical association and molecular mechanism levels avoids the limitations of single clinical or basic research and enhances the reliability and comprehensiveness of the research results. ② By integrating the gene expression data of osteoporosis and stroke in the GEO database, three new common key genes, LILRA5, HNRNPL, and AGBL3, were screened out, and their diagnostic efficacy for the two diseases was verified (AUC mostly greater than 0.8). Meanwhile, three potential intervention drugs, Cyclopenthiazide, Neostigmine bromide and R-atenolol, were predicted and verified through molecular conjugation, providing new candidate targets for the development of diagnostic markers and drug treatment of osteoporosis – stroke comorbidity. ③ Through functional enrichment analysis, it was found that the common key genes were mainly enriched in biological processes such as “RNA splicing regulation” and “metallopeptidase activity”, as well as signaling pathways such as “spliceosome”, “mRNA surveillance pathway”, and “amyotrophic lateral sclerosis (ALS) pathway”. A new mechanism of “RNA splicing disorder - inflammation - oxidative stress” synergistically regulating the comorbidity of osteoporosis and stroke was proposed, providing a new theoretical perspective for understanding the comorbidity nature of the two diseases.

However, this study has some limitations. First, the cross-sectional design of NHANES cannot establish causal temporal relationships between Osteoporosis and stroke, nor can it rule out the influence of residual confounding factors (such as bone turnover markers, vitamin D levels); second, the NHANES data lack objective examination imaging data for stroke, making it impossible to verify the consistency between self-reported diagnosis and the objective indicators in the NHANES data; third, the drug predictions and molecular docking from DSigDB are only computer simulations and have not yet been validated for their activity, toxicity, and bone-vascular dual-target dose-pharmacokinetic characteristics through cellular or animal experiments, necessitating subsequent assessments of safety windows and efficacy using organoid-chip and in vivo model systems.

## 5. Conclusion

Our study provides further evidence supporting a positive association between Osteoporosis and Stroke risk, which aligns with the trends observed in previous relevant research. Through bioinformatics analysis, LILRA5, HNRNPL, and AGBL3 were identified as potential common key genes for both diseases. Additionally, Cyclopenthiazide, Neostigmine bromide, and R-atenolol were predicted to potentially modulate these three genes, though this regulatory effect requires further experimental validation. These findings may offer new insights for exploring the prevention and treatment strategies of comorbidity between Osteoporosis and stroke.

## Supporting information

S1 File Comparison of population before and after PSM analysis. (DOCX)
