# Supplementary material for: Exploring the link between osteoporosis and stroke risk: An exploratory study based on 2017–2018 NHANES clinical data and bioinformatics analysis
Source: PLoS One. 2025 Dec 11;20(12):e0337460. doi: 10.1371/journal.pone.0337460 (PMC12697941; doi:10.1371/journal.pone.0337460)
Supplement: S1 File — (DOCX) [file pone.0337460.s001.docx]

**Supplementary Table**

| Supplementary Table 1. Comparison of population before and after PSM analysis | | | | | | | | | | |
| --- | --- | --- | --- | --- | --- | --- | --- | --- | --- | --- |
|  | Before PSM Aanalysis | | | | | After PSM analysis | | | | |
| Variable | Total (n = 2585) | No-Osteoporosis (n = 2256) | Osteoporosis (n = 329) | *P-value* | SMD | Total (n = 951) | No-Osteoporosis (n = 622) | Osteoporosis (n = 329) | *P-value* | SMD |
|  |  |  |  |  |  |  |  |  |  |  |
| Age, Mean ± SD | 64.95 ± 9.12 | 64.30 ± 9.01 | 69.42 ± 8.63 | **<.001** | 0.593 | 69.25 ± 8.62 | 69.17 ± 8.62 | 69.42 ± 8.63 | 0.672 | 0.029 |
| BMI, (kg/m²), Mean ± SD | 29.86 ± 6.95 | 29.95 ± 6.88 | 29.25 ± 7.37 | 0.089 | -0.095 | 29.42 ± 7.05 | 29.50 ± 6.88 | 29.25 ± 7.37 | 0.606 | -0.034 |
| Gender, n (%) |  |  |  | **<.001** |  |  |  |  | 0.505 |  |
| Female | 1310 (50.68) | 1030 (45.66) | 280 (85.11) |  | 1.108 | 799 (84.02) | 519 (83.44) | 280 (85.11) |  | 0.047 |
| Male | 1275 (49.32) | 1226 (54.34) | 49 (14.89) |  | -1.108 | 152 (15.98) | 103 (16.56) | 49 (14.89) |  | -0.047 |
| Race, n (%) |  |  |  | **0.003** |  |  |  |  | 0.86 |  |
| Mexican American | 298 (11.53) | 270 (11.97) | 28 (8.51) |  | -0.124 | 78 (8.2) | 50 (8.04) | 28 (8.51) |  | 0.017 |
| Other hispanic | 259 (10.02) | 227 (10.06) | 32 (9.73) |  | -0.011 | 87 (9.15) | 55 (8.84) | 32 (9.73) |  | 0.03 |
| Non-hispanic white | 985 (38.1) | 830 (36.79) | 155 (47.11) |  | 0.207 | 436 (45.85) | 281 (45.18) | 155 (47.11) |  | 0.039 |
| Non-hispanic black | 601 (23.25) | 543 (24.07) | 58 (17.63) |  | -0.169 | 185 (19.45) | 127 (20.42) | 58 (17.63) |  | -0.073 |
| Other | 442 (17.1) | 386 (17.11) | 56 (17.02) |  | -0.002 | 165 (17.35) | 109 (17.52) | 56 (17.02) |  | -0.013 |
